# Ebola Transmission Linked to a Single Traditional Funeral Ceremony — Kissidougou, Guinea, December, 2014–January 2015

**Published:** 2015-04-17

**Authors:** Kerton R. Victory, Fátima Coronado, Sâa O. Ifono, Therese Soropogui, Benjamin A. Dahl

**Affiliations:** 1 Epidemic Intelligence Service, CDC; 2 Division of Surveillance, Hazard Evaluations, and Field Studies, National Institute for Occupational Safety and Health, CDC; 3 Office of Public Health Scientific Services, Center for Surveillance, Epidemiology, and Laboratory Services, CDC; 4 World Health Organization, Kissidougou, Guinea; 5 Guinea Ministry of Health, Kissidougou; 6 Global Immunization Division, Center for Global Health, CDC

On December 18, 2014, the Guinea Ministry of Health was notified by local public health authorities in Kissidougou, a prefecture in southeastern Guinea (pop. 284,000), that the number of cases of Ebola virus disease (Ebola) had increased from one case reported during December 8–14, 2014, to 62 cases reported during December 15–21. Kissidougou is one of the four Guinea prefectures (the others are Macenta, Gueckedou, and Conakry) where Ebola was first reported in West Africa in March 2014 (1), and the mid-December increase was the largest documented by any prefecture in Guinea in a single week since the beginning of the epidemic. The Guinea Ministry of Health requested assistance from CDC and the World Health Organization to investigate the local outbreak, identify and isolate persons with suspected Ebola, assess transmission chains, and implement control measures. The investigation found that 85 confirmed Ebola cases were linked to one traditional funeral ceremony, including 62 (73%) cases reported during December 15–21. No additional cases related to this funeral ceremony were reported after January 10, 2015. After the outbreak was identified, rapid implementation of interventions limited additional Ebola virus transmission. Improved training for prompt reporting of cases, investigation, and contact tracing, and community acceptance of safe burial methods can reduce the risk for Ebola transmission in rural communities.

## Epidemiology and Laboratory Testing

On December 19, 2014, rapid response teams including epidemiologists, clinicians, and local public health officials were deployed to villages where potential cases had been reported. The teams interviewed patients and household contacts and conducted active identification of cases and contacts. Ebola case investigation forms were reviewed to identify and characterize cases reported during December 1, 2014–January 10, 2015 as either suspected, probable, or confirmed. A suspected case was defined as one with Ebola-compatible symptoms (i.e., fever and malaise with other nonspecific signs and symptoms, including myalgia, headache, vomiting, and diarrhea) in a Kissidougou resident; a probable case was defined as Ebola-compatible symptoms reported for a decedent for whom no specimens were collected; and a confirmed case was defined as Ebola-compatible symptoms in a person with ≥1 Ebola virus–positive specimen tested by reverse transcription–polymerase chain reaction (RT-PCR) (2). Patients with suspected Ebola were isolated and transported to an Ebola treatment center (ETC) for confirmation of Ebola virus by RT-PCR. For decedents with suspected Ebola, oral swabs were collected within 24 hours upon notification of death, and the swabs were sent to an ETC for confirmation of Ebola.

Specimens from 62 persons tested positive for Ebola virus by RT-PCR. Review of case investigation forms and reports indicated that all 62 confirmed cases lived in Kissidougou and were clustered in six villages: 29 (47%) in Ouendero, 13 (21%) in Kamandou, eight (13%) in Mandou, five (8%) in Kongola, four (6%) in Tangolto, and three (5%) in Gbeninkoro. Thirty-two (52%) of the patients were male. Median age was 35 years (range = 2–80 years); four (6%) patients were aged <15 years, 37 (60%) were aged 15–49 years, and 21 (34%) were aged ≥50 years.

Fifty-six (90%) of the 62 patients had Ebola-compatible symptoms. Fifty-one (82%) died; 33 (65%) died in an ETC, and 18 (35%) decedents were reported as community deaths. These community deaths occurred during December 14–17 in three villages in Kissidougou: Mandou (seven deaths), Kamandou (six), and Ouendero (five). Patients who died in the community had not sought medical treatment; instead, family members reported the deaths to local health authorities, who considered them as suspected Ebola cases. Oral swabs were collected from all 18 decedents within 24 hours upon notification of death; all tested positive for Ebola virus and were reclassified as confirmed Ebola cases.

## The Funeral of the Midwife Assistant

Interviews with household contacts of the 18 decedents reported from the community revealed that they all occurred in persons who had attended the funeral ceremony in early December of a well-known local male midwife assistant (patient 1) who regularly performed circumcisions in the community. Patient 1 had traveled from Ouendero to Djomakoidou, a village 3 hours away in Macenta, to perform a circumcision on an infant in mid-November 2014; a villager reported that the child subsequently died of an unknown cause. Approximately 1 week after he returned to Ouendero, patient 1 reportedly became ill with Ebola-compatible symptoms and died on December 4, 2014. However, he did not seek medical attention, and the cause of his death was reported as unknown. His funeral ceremony on December 4, 2014 was attended by approximately 100 persons from Ouendero and neighboring villages. Traditional burial practices in Guinea and other West African countries typically involve washing, touching, and kissing of the body of the deceased; therefore, it is likely that several attendees could have had direct contact with the body and body fluids. On December 18, 2014, patient 1 was classified as having probable Ebola.

What is already known on this topic?

Ebola can be transmitted through direct contact with the corpse or body fluids of an infected person, including during traditional funeral ceremonies.

What is added by this report?

During December 1, 2014–January 10, 2015, an outbreak of 85 confirmed Ebola cases was linked to one traditional funeral ceremony in Kissidougou, Guinea. Eighteen (21%) of the 85 with confirmed infection attended the funeral and had direct contact with the corpse, and 67 (79%) had direct contact with at least one attendee of the funeral.

What are the implications for public health practice?

Training in and adherence to hygienic burial of corpses infected with Ebola and community acceptance of culturally sensitive safe burial practices is an important component of the successful management of Ebola and prevention of further transmission.

**FIGURE 1:**
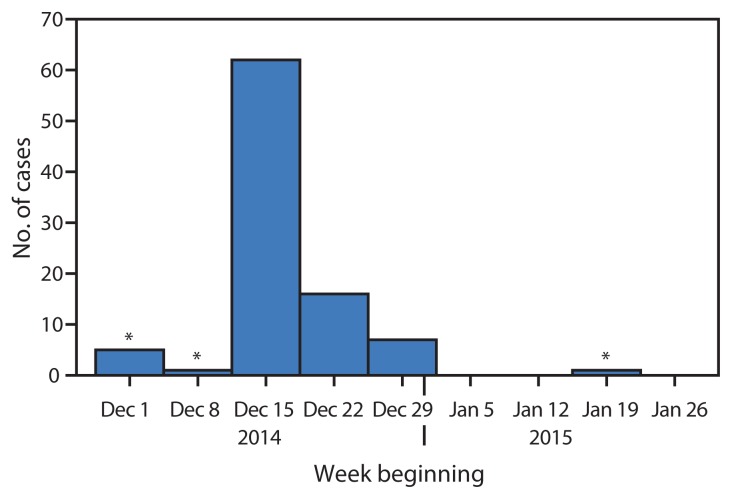
Number of confirmed Ebola cases, by week — Kissidougou, Guinea, December, 2014–January 2015 * Cases not related to the midwife assistant’s funeral cemerony.

**FIGURE 2:**
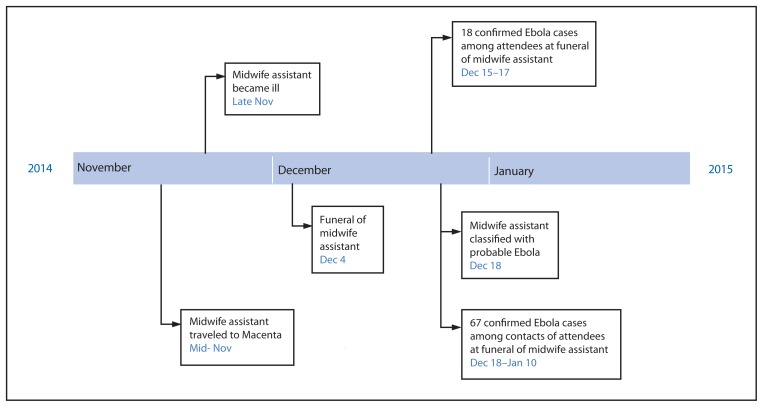
Timeline of events regarding Ebola cases linked to a single funeral ceremony — Kissidougou, Guinea, November, 2014–January 2015

As of January 31, 2015, a total of 85 confirmed Ebola cases were linked to this one traditional funeral ceremony, including 62 (73%) cases reported during December 15–21 (Figure 1, Figure 2). Eighteen (21%) Ebola patients attended the funeral and had direct contact with the body of patient 1, and 67 (79%) had direct contact with at least one attendee of the funeral. Forty-one (48%) patients were male; median age was 33 years (range = 2–85 years). Sixty-three of the 85 patients with confirmed Ebola died (case-fatality rate = 74%). No additional cases related to this funeral ceremony were reported after January 10. Additionally, a total of 780 contacts were monitored in 12 villages by nine contact-tracing teams for 21 days following their last potential exposure. However, this effort might not have covered all contacts. Local public health authorities reported that they were not allowed to enter some villages and identify all contacts because of mistrust and resistance in several communities.

### Discussion

This investigation encountered challenges associated with responding to the Ebola epidemic in Guinea, including incomplete ascertainment, reporting, and investigation of cases; unsafe burial practices; and community reticence, particularly in remote areas. To control Ebola transmission in Kissidougou and other difficult-to-reach communities in Guinea, targeted involvement of community leaders and enhancement of public health interventions are crucial for the proper implementation of Ebola prevention and control strategies. These enhancements include 1) educating the community regarding the signs and symptoms of Ebola and its modes of transmission, 2) stressing the importance of seeking medical care and reporting suspected Ebola cases, and 3) emphasizing the potential benefit of early diagnosis and treatment. Targeted education strategies and health communication messages in local languages can help decrease the concerns of groups resistant to the Ebola intervention efforts of local public health officials (3) and can facilitate the isolation and limited treatment of patients who are unwilling or unable to seek care at an ETC (4).

This investigation also revealed that although mechanisms have been recommended for transporting persons with suspected Ebola to the nearest ETCs, intrinsic challenges of transportation in rural communities (i.e., poor transportation and communication infrastructure) remain a major problem. In Kissidougou, patients were transferred to one of the nearest ETCs in either Gueckedou (52 miles [2-hour drive]) or Macenta (83 miles [3-hour drive]), which delayed the time from identification to isolation, diagnosis, and treatment at an ETC, and created the potential for exposure of additional persons. Safe transportation support to link persons with suspected Ebola to treatment centers should be facilitated immediately after the cases are reported to health authorities. Special strategies such as implementation of communication plans to alert local public health authorities and deployment of rapid response teams have been shown to be very effective, especially in rural areas (4,5).

Ebola can be transmitted through direct contact with the corpse or body fluids of an infected person, especially during traditional funeral ceremonies. As evidenced by this investigation, these exposures can result in outbreaks when there are obstacles to educating populations on adequate public health interventions. Improved training in hygienic burial of dead bodies and community acceptance of culturally sensitive safe burial are needed to ensure successful management of Ebola cases and prevent further transmission (6).

The findings of this investigation highlight the importance of controlling local outbreaks in difficult-to-reach communities as a key component of the effort to eliminate Ebola (5). Although public health interventions were established before this local outbreak, they were not fully implemented in Kissidougou, where they could have prevented or reduced Ebola transmission at the funeral ceremony. After the outbreak was identified, rapid implementation of interventions limited further Ebola virus transmission.
